# Evaluation of novel fungicides (FRAC groups 7, 9, 12) for managing cranberry fruit rot

**DOI:** 10.3389/fpls.2024.1508744

**Published:** 2025-02-10

**Authors:** Leela Saisree Uppala, Salisu Sulley

**Affiliations:** University of Massachusetts-Amherst Cranberry Station, Wareham, MA, United States

**Keywords:** CFR, cranberry fruit rot, chlorothalanil, fruit rot management, Massachusetts cranberries

## Abstract

Cranberry fruit rot (CFR) is a major disease complex that significantly impacts cranberry crops, leading to substantial yield losses. Over the past decade, CFR has become increasingly problematic, particularly in high-yielding and newer cultivars, with reported losses ranging from 50% to 100%. Additionally, the cranberry industry faces increasing restrictions on the use of broad-spectrum fungicides, such as chlorothalonil and mancozeb, necessitating the exploration of alternative management strategies. This study, conducted from 2021 to 2024 at the University of Massachusetts-Amherst Cranberry Station, evaluated novel fungicides from FRAC Groups 7, 9, and 12. The active ingredients—benzovindiflupyr, pydiflumetofen, cyprodinil, and fludioxonil—were tested individually and in combination with azoxystrobin (FRAC 11). The efficacy of these fungicides in reducing CFR incidence and improving yield was assessed on cranberry cultivars ‘Demoranville’, ‘Ben Lear,’ and ‘Stevens’ with applications made at early, mid, and late bloom stages. Significant differences in fruit rot incidence and yield were observed in 2021, 2023 and 2024. Treatments containing pydiflumetofen, pydiflumetofen & fludioxonil, and benzovindiflupyr, when applied in combination with azoxystrobin, consistently resulted in lower rot incidence and higher yields. The treatment containing cyprodinil & fludioxonil plus azoxystrobin, tested only in 2021, also resulted in lower rot incidence and higher yield. These findings highlight the potential of novel fungicides from FRAC Groups 7, 9, and 12 as effective alternatives for CFR management. Their use could diversify the CFR management toolkit, mitigate fungicide resistance, and reduce environmental impacts, addressing the challenges posed by increasing fungicide regulations.

## Introduction

The cranberry fruit rot (CFR) a term used to describe general softening and deterioration of cranberries, affects the fruits both during preharvest and postharvest stages, significantly impacting the quality of cranberries intended for fresh-market consumption and processing (Oudemans et al., 1998). Research on fruit rot, dating back to the early 1900s, has highlighted the involvement of numerous taxonomically diverse fungal species, emphasizing the complexity of the disease (Bain, 1926; Stevens, 1924, 1932; Caruso et al., 2000; Conti et al., 2021; McManus et al., 2003; Olatinwo et al., 2004; Vilka and Bankina, 2013; Zuckerman, 1958).

The distribution of CFR fungal species, along with the incidence and severity of the resulting fruit rot, varies across cultivars, geographical locations, growing seasons, and phenological stages (Stevens, 1924; Bergman and Wilcox, 1936; Zuckerman, 1958; McManus et al., 2003; Oudemans et al., 1998; Polashock et al., 2009; Wells-Hansen and McManus, 2017). Literature suggests that 10–15 fungal species are most prevalent across growing regions. These include *Colletotrichum fioriniae* (Marcelino & Gouli ex R.G. Shivas & Y.P. Tan; teleomorph *Glomerella acutata* var. *fiorinae*), *Colletotrichum fructivorum* (V.P. Doyle, P.V. Oudem. & S.A. Rehner; teleomorph *Glomerella cingulata*), *Coleophoma empetri* Rostr., *Phyllosticta vaccinii* (Earle), *Physalospora vaccinii* Shear (Arx & E. Müll.), *Allantophomopsis lycopodina* (Höhn) Carris, *Allantophomopsis cytisporea* (Fr.) Petr., *Strasseria geniculata* (Berk. & Broome) Höhn, *Phyllosticta elongata* Weid. (teleomorph *Botryosphaeria vaccinii*), *Fusicoccum putrefaciens* Shear (teleomorph *Godronia cassandrae*), *Monilinia oxycocci* (Woronin) Honey, and *Phomopsis vaccinii* Shear (teleomorph *Diaporthe vaccinii*). Each species is associated with specific fruit rot diseases—bitter rot, ripe rot, early rot, blotch rot, black rot, berry speckle, end rot, cotton ball, and viscid rot—collectively known as the CFR disease complex (McManus et al., 2003; Oudemans et al., 1998; Polashock et al., 2009; Raj, 1983; Weir et al., 2012; McManus, 2001). Our evaluations during the 2021 and 2022 growing seasons identified *C. empetri*, *A. cytisporea*, *B. vaccinii*, *P. vaccinii*, and various *Colletotrichum* species as the most prevalent fungal species in Massachusetts (unpublished data).

Cranberry handlers (the processing industry) often heavily discount deliveries with over 12% fruit rot, whereas those exceeding 20% are typically rejected outright. Consequently, potential yield losses due to CFR vary widely, spanning from 20% to 100% if not managed strategically (McManus et al., 2003; Olatinwo et al., 2004; Oudemans et al., 1998; Stiles and Oudemans, 1999; Wells et al., 2014; Wells-Hansen and McManus, 2017).

Currently, cranberry growers in Massachusetts and New Jersey rely on “in bloom” (during flowering) application of 3-5 fungicides for CFR management (Caruso et al., 2000; Oudemans et al., 1998). Despite fungicide use, fruit rot in these two states ranges from less than 1% up to 15% (Oudemans et al., 1998). Along with decreasing the fungal pathogen inoculum in the field (Averill et al., 2008), fungicide applications significantly increase the production costs and the risk of fungicide resistance development (Hahn, 2014; Lucas et al., 2015).

Further complicating this situation, in the past six years, there are increasing restrictions imposed by key export markets [(European Union (EU)], on the use of broad-spectrum fungicides (mancozeb and chlorothalonils) owing to perceived environmental and health risks. These fungicides have been the backbone of CFR management and fungicide resistance management for the past 50 years (Caruso et al., 2000). In 2019, the European Union (EU) published their decision to lower the Maximum Residue Limit (MRL) for chlorothalonil to 0.01 ppm (parts per million) from 5 ppm for cranberry fruit/processed products destined for the EU market. Additionally, the EU announced a non-renewal of mancozeb beginning in 2021, with a risk based MRL review pending. These regulatory constraints caused a significant shift in fungicide use patterns in Massachusetts. Commercial fungicides are categorized by the Fungicide Resistance Action Committee (FRAC, 2020) based on their target site, chemical class, and risk of resistance development (Brent and Hollomon, 1998). Most growers are now relying on other classes of fungicides (single-site mode of action) from FRAC (Fungicide Resistance Action Committee) Group 3 (systemic demethylation inhibitors- DMIs) with active ingredients (a.i) prothioconazole (Proline^®^), fenbuconazole (Indar^®^); and Group 11 (strobilurins- a group of QoI -Quinone Outside Inhibitor fungicides) with a.i. azoxystrobin (Abound^®^). Although these newer classes of fungicides are highly effective and less harmful to human and environmental health, they pose a much higher risk of selecting for fungicide resistance compared to broad-spectrum fungicides. Resistance to these groups of fungicides has been well documented across several cropping systems (Ding et al., 2019; Hunter et al., 1984; Kikuhara et al., 2019; Rosenzweig et al., 2019; Schnabel et al., 2004; Trueman et al., 2017; Wolfe, 1985; Yamaguchi et al., 2000).

For the long-term sustainability of the cranberry industry, it is essential to diversify the fruit rot management tool kit. In our search for novel fungicide options, we identified several promising novel groups of fungicides that were reported to have effectiveness against a broad spectrum of fungal pathogens across various crops. These include succinate dehydrogenase inhibitors (SDHIs), such as benzovindiflupyr and pydiflumetofen, cyprodinil fungicides from the anilino-pyrimidine group (FRAC Group 9), and fludioxonil from the phenylpyrrole group (FRAC Group 12) (Ayer et al., 2021; Ishii et al., 2016; Wedge et al., 2007; Thomidis and Prodromou, 2018; Xiao and Boal, 2009; Mouden et al., 2016; Forster et al., 2007). Each fungicide exhibits a distinct mode of action. For instance, SDHIs target succinate dehydrogenase (SDH), a critical mitochondrial enzyme essential for energy production in eukaryotic organisms (Hospital et al., 2023). Similarly, anilino-pyrimidine fungicides, such as cyprodinil, inhibit methionine biosynthesis and the secretion of hydrolytic enzymes, thereby disrupting fungal development (Mosbach et al., 2017). These diverse mechanisms help reduce the risk of fungicide resistance. Thus the current study was initiated with an objective to evaluate the efficacy of selected fungicides from FRAC Groups 7 (containing benzovindiflupyr and pydiflumetofen), 9 (containing cyprodinil), 12 (containing fludioxonil) on fruit rot incidence and fruit yield.

## Materials and methods

### Evaluation of selected novel fungicides for CFR disease management

#### Experimental design

Fungicide evaluation trials were conducted in 2021-2024 at the University of Massachusetts-Amherst Cranberry Station in East Wareham. The fungicides and their application rates evaluated in this study are detailed in **Table 1**. In 2021 and 2023, experiments were conducted on cranberry cultivar ‘Demoranville’, whereas in 2022 and 2024 the experiments were conducted in ‘Ben Lear’ and ‘Stevens’ respectively, due to the unavailability of “Demoranville” cultivar. Anecdotally, ‘BenLear’ and ‘Demoranville’ cultivars are known to have lowest field rot resistance; while the cultivar ‘Stevens’ is known to have moderate field rot resistance (Uppala and Sylvia, 2024). In all trials, treatments were applied to 1-m^2^ plots with 0.6-m-wide nontreated borders between the plots, serving as buffer zones. In all trials, the experimental design was a randomized complete block design (RCBD) with five replications. Sprays were applied at designated phenological stages using a CO_2_ backpack sprayer equipped with a single flat-fan nozzle (Tee-Jet 8004) at 275.79 Kilopascals (kPa) or 40 psi at the rate of 2806 liters/hectare (300 gallons/acre). Applications were performed in low-wind conditions to minimize drift between plots.

**Table 1:** Fungicides evaluated for the management of cranberry fruit rot in this study.

Brand name	Active ingredients	Company	FRAC Code	Application rate
Abound^®^ Flowable	Azoxystrobin	Syngenta	11	1.13 l/ha
Aprovia^®^	Benzovindiflupyr	Syngenta	7	0.77 l/ha
ManzateMax^®^	Zinc, Manganese & EBDC	UPL	M3	5.38 kg/ha
Miravis^®^	Pydiflumetofen	Syngenta	7	0.78 l/ha
MiravisPrime^®^	Pydiflumetofen & Fludioxonil	Syngenta	7 & 12	0.83 l/ha
Proline^®^ 480 SC	Prothioconazole	Bayer	3	0.37 l/ha
QuadrisTop^®^	Difenoconazole & Azoxystrobin	Syngenta	3 & 11	1.02 l/ha
Switch^®^ 62.5 WG	Cyprodinil & Fludioxonil	Syngenta	9 & 12	0.91 l/ha

FRAC, Fungicide Resistance Action Committee.

EBDC, Ethylenebisdithiocarbamate.

#### Fungicide application regimes

The spray schedule for all the growing seasons and details of the fungicide products evaluated are provided in **Table 2**–**5**. Dates of fungicide application sprays were provided in **Table 6** Briefly, in 2021, novel fungicides each with active ingredients “cyprodinil & fludioxonil” (FRAC 9 & 12), benzovindiflupyr (FRAC 7), “pydiflumetofen & fludioxonil” (FRAC 7 & 12) were evaluated by themselves; or with azoxystrobin (FRAC 11) in three spray regimes: early bloom (EB- when 30% flowers were open), mid-bloom (MB- 7-14 days after the previous application), and late bloom (LB- 7-14 days after the previous application). The efficacy of these treatments was compared with non-sprayed control, and a treatment with registered products QuadrisTop^®^ (a.i. difenoconazole & azoxystrobin) applied at EB & MB followed by Proline^®^ (prothioconazole) applied at LB (**Table 2**).

**Table 2:** Effect of fungicide application on the fruit rot and yield of cranberry, 2021.

Spray Schedule			Percent Fruit Rot	Yield (kg/ha)
Spray 1	Spray 2	Spray 3		
Non-Sprayed	Non-Sprayed	Non-Sprayed	48 ab^Z^	3777 e
Difenoconazole & Azoxystrobin	Difenoconazole & Azoxystrobin	Prothioconazole	52 a	7631 cd
Cyprodinil & Fludioxonil	Cyprodinil & Fludioxonil	Cyprodinil & Fludioxonil	52 a	4656 de
Pydiflumetofen & Fludioxonil	Pydiflumetofen & Fludioxonil	Pydiflumetofen & Fludioxonil	38 abc	10218 bc
Benzovindiflupyr	Benzovindiflupyr	Benzovindiflupyr	50 ab	5526 de
Cyprodinil & Fludioxonil + Azoxystrobin	Cyprodinil & Fludioxonil + Azoxystrobin	Cyprodinil & Fludioxonil + Azoxystrobin	25 cd	13549 a
Pydiflumetofen & Fludioxonil + Azoxystrobin	Pydiflumetofen & Fludioxonil + Azoxystrobin	Pydiflumetofen & Fludioxonil + Azoxystrobin	23 d	11226 ab
Benzovindiflupyr + Azoxystrobin	Benzovindiflupyr + Azoxystrobin	Benzovindiflupyr + Azoxystrobin	38 bc	7302 cd

z Means within a column followed by the same letter are not significantly different according to Fisher’s Protected LSD (P<0.05).

**Table 3:** Effect of fungicide application on the fruit rot and yield of cranberry, 2022.

Spray Schedule			Percent Fruit Rot	Yield (kg/ha)
Spray 1	Spray 2	Spray 3		
Non-Sprayed	Non-Sprayed	Non-Sprayed	29	16199
Zinc, Manganese & EBDC	Difenoconazole & Azoxystrobin	Prothioconazole	21	16343
Azoxystrobin	Azoxystrobin	Azoxystrobin	25	17134
Difenoconazole & Azoxystrobin	Difenoconazole & Azoxystrobin	Difenoconazole & Azoxystrobin	32	17564
Pydiflumetofen	Pydiflumetofen	Pydiflumetofen	22	15054
Pydiflumetofen & Fludioxonil	Pydiflumetofen + Fludioxonil	Pydiflumetofen + Fludioxonil	22	17273
Benzovindiflupyr	Benzovindiflupyr	Benzovindiflupyr	25	18885
Pydiflumetofen + Azoxystrobin	Pydiflumetofen + Azoxystrobin	Pydiflumetofen +Azoxystrobin	17	22216
Pydiflumetofen & Fludioxonil + Azoxystrobin	Pydiflumetofen & Fludioxonil + Azoxystrobin	Pydiflumetofen & Fludioxonil + Azoxystrobin	14	19615
Benzovindiflupyr + Azoxystrobin	Benzovindiflupyr + Azoxystrobin	Benzovindiflupyr + Azoxystrobin	17	23994

**Table 4:** Effect of fungicide application on the fruit rot and yield of cranberry, 2023.

Spray Schedule			Percent Fruit Rot	Yield (kg/ha)
Spray 1	Spray 2	Spray 3		
Non-Sprayed	Non-Sprayed	Non-Sprayed	31 a^z^	13567 c
Zinc, Manganese & EBDC	Difenoconazole & Azoxystrobin	Prothioconazole	13 cde	24930 ab
Azoxystrobin	Azoxystrobin	Azoxystrobin	26 ab	22415 ab
Benzovindiflupyr	Benzovindiflupyr	Benzovindiflupyr	24 b	17275 bc
Benzovindiflupyr + Azoxystrobin	Benzovindiflupyr + Azoxystrobin	Benzovindiflupyr + Azoxystrobin	23 b	27912 a
Difenoconazole & Azoxystrobin	Difenoconazole & Azoxystrobin	Difenoconazole & Azoxystrobin	16 c	22309 ab
Pydiflumetofen	Pydiflumetofen	Pydiflumetofen	15 cd	24768 ab
Pydiflumetofen & Fludioxonil	Pydiflumetofen & Fludioxonil	Pydiflumetofen & Fludioxonil	15 cd	23000 ab
Pydiflumetofen + Azoxystrobin	Pydiflumetofen + Azoxystrobin	Pydiflumetofen + Azoxystrobin	10 de	27780 a
Pydiflumetofen & Fludioxonil + Azoxystrobin	Pydiflumetofen & Fludioxonil + Azoxystrobin	Pydiflumetofen & Fludioxonil + Azoxystrobin	8 e	23746 ab

^z^Means within a column followed by the same letter are not significantly different according to Fisher’s Protected LSD (P<0.05).

**Table 5:** Effect of fungicide application on the fruit rot and yield of cranberry, 2024.

Spray Schedule			Percent Fruit Rot	Yield (kg/ha)
Spray 1	Spray 2	Spray 3		
Non-Sprayed	Non-Sprayed	Non-Sprayed	55 a^z^	8454 c
Difenoconazole & Azoxystrobin	Zinc, Manganese & EBDC	Prothioconazole	12 d	15667 abc
Azoxystrobin	Azoxystrobin	Azoxystrobin	25 bcd	17002 ab
Benzovindiflupyr	Benzovindiflupyr	Benzovindiflupyr	34 bc	18817 a
Benzovindiflupyr + Azoxystrobin	Benzovindiflupyr + Azoxystrobin	Benzovindiflupyr + Azoxystrobin	13 d	22819 a
Pydiflumetofen	Pydiflumetofen	Pydiflumetofen	47 ab	10104 bc
Pydiflumetofen & Fludioxonil	Pydiflumetofen & Fludioxonil	Pydiflumetofen & Fludioxonil	36 abc	15749 abc
Pydiflumetofen + Azoxystrobin	Pydiflumetofen + Azoxystrobin	Pydiflumetofen + Azoxystrobin	24 cd	15468 abc
Pydiflumetofen & Fludioxonil + Azoxystrobin	Pydiflumetofen & Fludioxonil + Azoxystrobin	Pydiflumetofen & Fludioxonil + Azoxystrobin	24 cd	21036 a

z Means within a column followed by the same letter are not significantly different according to Fisher’s Protected LSD (P<0.05).

**Table 6:** Spray application dates for each of the fungicide trials.

Fungicide Spray Application Dates			
Study Year	Early Bloom	Mid Bloom	Late Bloom
2021	06/19	07/02	07/13
2022	06/16	06/28	07/06
2023	06/20	06/29	07/09
2024	06/14	06/25	07/08

In 2022, 2023 and 2024, novel fungicides with active ingredients benzovindiflupyr, pydiflumetofen, “pydiflumetofen & fludioxonil” were evaluated by themselves; or in combination with azoxystrobin in three spray regimes. The efficacy of these treatments was compared with non-sprayed control, and a treatment with registered products Zinc, Manganese & EBDC applied at EB, QuadrisTop^®^ (a.i. difenoconazole & azoxystrobin) applied at MB followed by Proline^®^ (prothioconazole) applied at LB. This application regime was named as the grower standard.

#### Sample c*ollection, disease and yield assessment*

At harvest maturity (late September to early October), berries from within an arbitrarily selected 0.3-m^2^ area of each replicated plot were harvested by hand. From each sample, fruits were passed through a 5.6 mm opening/mesh size sieve. The berries that passed through the sieve were discarded as pinheads. The fruits that were retained on the top (industry standard sized fruits) were sorted into 2 categories: healthy and rotten berries. Rotten berries were counted to determine the fruit rot incidence (% Rot) which was calculated as the proportion of rotten berries to the total number of berries, expressed as a percentage.

Yield data were obtained by converting the weight of the healthy berries from each 0.3-m^2^ area to kg/ha. Weather summaries (Mean monthly temperature, relative humidity; Monthly number of rainy days and total precipitation) were compiled from visual crossing website (https://www.visualcrossing.com/weather-data) and presented in **Table 7**.

**Table 7:** Summary of weather data collected during fungicide trials in East Wareham, Massachusetts.

Year	Month	Mean Temperature (°C)	Mean Relative Humidity (%)	Total Precipitation (mm)	No. of rainy days
**2021**	June	21	74	55	13
	July	21	82	163	22
	August	23	80	65	11
	September	19	80	279	15
**2022**	June	19	72	87	11
	July	24	69	19	10
	August	24	77	141	11
	September	18	79	83	11
**2023**	June	18	84	112	17
	July	24	84	101	13
	August	21	81	104	17
	September	19	84	159	14
**2024**	June	21	71	60	12
	July	24	76	38	15
	August	22	77	84	11
	September	18	77	110	8

Weather data were downloaded from visual crossing website (https://www.visualcrossing.com/weather-data) for East Wareham, MA, USA, Zip code: 02538.

### Data analysis

Fungicide trials data were subjected to analysis of variance (ANOVA) using lme4 package using Mixed Model analysis in R (R Core Team, 2024) to determine the effects of fungicide treatments on percent CFR incidence (% Rot) and yield. Replications were considered random effects. The data were examined for normal distributions and homoscedasticity using the Shapiro Wilk and Barlett tests, respectively. Logarithmic transformations were performed when data did not fit a normal distribution. After statistical analysis, the means were back transformed to their original units. Treatment means were differentiated using Fisher’s LSD test, with alpha set to 0.05 and adjusted with the Bonferroni correction for multiple comparisons.

## Results

### Effect of fungicides on fruit rot and yield

Significant differences in percent fruit rot between treatments were observed in two of the three study years (P = 0.0003 in 2021; P = 0.0002 in 2023 and P = 0.014 in 2024). However, no significant differences were found in 2022 (P = 0.1). Similarly, significant differences in yield were observed in 2021 (P = 0.00005), 2023 (P = 0.03) and 2024 (P = 0.011), but not in 2022 (P = 0.34).

In 2021, fungicide treatments showed varied effects on cranberry fruit rot and yield. Plots treated with the combination of “cyprodinil & fludioxonil” plus azoxystrobin and “pydiflumetofen & fludioxonil” plus azoxystrobin had the lowest rot incidence levels, at 25% and 23%, respectively (**Table 2**). These were followed by plots treated with “benzovindiflupyr plus azoxystrobin” and the combination of “pydiflumetofen & fludioxonil,” which resulted in an average rot incidence of 38%. All other treatments resulted in rot levels similar to the non-sprayed control, with an average of 48% rot. In terms of yield, the treatments with the lowest rot levels (“cyprodinil & fludioxonil + azoxystrobin” and “pydiflumetofen & fludioxonil + azoxystrobin”) also resulted in the highest average yields, at 12,388 kg/ha. These were followed by “pydiflumetofen & fludioxonil” alone (10,218 kg/ha) and “benzovindiflupyr plus azoxystrobin” (7,302 kg/ha). The non-sprayed control, as well as treatments with cyprodinil & fludioxonil and benzovindiflupyr alone (without azoxystrobin), resulted in the lowest average yields, at 4,653 kg/ha.

In 2022, rot incidence was generally lower than in 2021. While the treatments did not reach significance at the 5% level, it is noteworthy that they were significant at the 10% level. Similar to 2021, “pydiflumetofen & fludioxonil + azoxystrobin” resulted in the lowest rot incidence (**Table 3**), comparable to treatments with “pydiflumetofen” and “benzovindiflupyr” applied with azoxystrobin, with an average rot level of 16%. All other treatments resulted in higher rot levels (average of 25%), similar to the non-sprayed control (**Table 3**). Yield-wise, there were no significant differences between treatments, and the average yield was 18,428 kg/ha.

In 2023, fungicide treatments continued to show varying levels of efficacy in controlling fruit rot (**Table 4**). The application of “pydiflumetofen & fludioxonil + azoxystrobin” resulted in the lowest rot incidence (8.4%), which was comparable to treatments with “pydiflumetofen + azoxystrobin” and the grower standard. Significantly higher rot levels were observed in the non-sprayed control and in plots treated with azoxystrobin alone. Yield-wise, all fungicide treatments resulted in significantly higher yields compared to the non-sprayed control (13,567 kg/ha). Plots treated with benzovindiflupyr yielded 17,275 kg/ha, whereas all other treatments had comparable yields, averaging 24,608 kg/ha.

In 2024, fungicide treatments continued to exhibit varying levels of efficacy in controlling fruit rot. The highest fruit rot incidence was observed in the non-sprayed control (55%), while the lowest incidence was recorded for treatments with benzovindiflupyr + azoxystrobin (13%) and the grower standard (12%). Treatments with azoxystrobin alone and its combinations with pydiflumetofen or pydiflumetofen & fludioxonil showed intermediate efficacy, with rot levels averaging 24%. In contrast, pydiflumetofen and pydiflumetofen & fludioxonil treatments without azoxystrobin resulted in higher rot incidences of 47% and 36%, respectively. The highest yield was achieved in plots treated with benzovindiflupyr + azoxystrobin (22,819 kg/ha), highlighting its dual effectiveness in reducing rot and enhancing productivity. In comparison, the lowest yield was observed in non-sprayed plots (8,454 kg/ha).

## Discussion

Massachusetts growers have primarily relied on cultural control methods, such as late water flooding, pruning, and sanding, as well as chemical control options like chlorothalonils, mancozebs, copper-based fungicides, Quinone outside Inhibitors (FRAC group 11), and Demethylation Inhibitor (DMI) fungicides (FRAC group 3) to manage fruit rot and improve fruit quality. In a typical commercial setting, up to five fungicide applications are made per season, significantly raising cultivation costs. Besides the economic burden, growers have faced additional challenges in the past five years due to increased restrictions on broad-spectrum, multi-site fungicides such as mancozebs and chlorothalonils. These two groups of fungicides have been the backbone of cranberry fruit rot (CFR) and resistance management in Massachusetts (Caruso et al., 2000). Additionally, resistance to DMI and QoI fungicides has been well-documented across several cropping systems (Ding et al., 2019; Hunter et al., 1984; Kikuhara et al., 2019; Rosenzweig et al., 2019; Schnabel et al., 2004; Trueman et al., 2017; Wolfe, 1985; Yamaguchi et al., 2000).

Due to the complexity of the cranberry fruit rot, which involves multiple fungal pathogens, screening new fungicides and securing registrations requires significant time and resources. This has created an urgent need to diversify the existing CFR toolkit to reduce overreliance on current fungicides.

To address this, we evaluated the effectiveness of several novel broad-spectrum fungicide groups (FRAC 7, 9, 12), individually and in combination with azoxystrobin (FRAC 11). The active ingredients assessed included benzovindiflupyr and pydiflumetofen (FRAC 7), which are succinate dehydrogenase inhibitors that target the enzyme SDH in complex II of the mitochondrial respiration chain, disrupting the tricarboxylic acid cycle and electron transport chain (Keon et al., 1991). Cyprodinil (FRAC 9), an anilino-pyrimidine fungicide, inhibits methionine biosynthesis, germ tube elongation and hyphal growth (National Center for Biotechnology Information, 2025), whereas fludioxonil (FRAC 12), a phenylpyrrole fungicide, causes several physiological changes in pathogens, such as membrane hyperpolarization, changes in carbon metabolism, and metabolite accumulation, ultimately leading to hyphal swelling and bursting (Kilani and Fillinger, 2016). Azoxystrobin (FRAC 11), a quinone outside inhibitor, arrests fungal growth by inhibiting mitochondrial respiration at the Qo-site in complex III, thereby blocking electron transport (Becker et al., 1981).

Significant differences in fruit rot incidence and yield were observed among treatments and between growing seasons. Fungicides containing, pydiflumetofen, pydiflumetofen & fludioxonil + azoxystrobin, and benzovindiflupyr + azoxystrobin consistently resulted in lower rot incidence and higher yields. Cyprodinil & fludioxonil combined with azoxystrobin was evaluated only during the 2021 growing season, where it demonstrated significant efficacy in reducing fruit rot and enhancing yield. Wood et al. (2023) highlighted the role of benzovindiflupyr, cyprodinil, and fludioxonil in inhibiting the mycelial growth of CFR fungi *in vitro.* Similarly Wisconsin field trials (McManus and Perry, 2018, 2019) reported the efficacy of cyprodinil & fludioxonil, pydiflumetofen, and benzovindiflupyr in managing fruit rot through field trials. In contrast to our study, which evaluated three spray regimes (early, mid, and late bloom) of these compounds individually and in combination with azoxystrobin, McManus and Perry (2018, 2019) tested them in two spray regimes (full bloom and early fruit set). In our trials, only treatments tank-mixed with azoxystrobin (except pydiflumetofen) reduced rot levels effectively. Conversely, McManus and Perry (2018, 2019) observed significantly lower rot levels when these products were applied individually. In the 2022 growing season, azoxystrobin applied alone in three spray regimes did not reduce rot compared to the non-sprayed control. However, azoxystrobin co-applied with pydiflumetofen, benzovindiflupyr, and pydiflumetofen & fludioxonil significantly lowered fruit rot, suggesting a synergistic effect.

Fruit rot levels across treatments ranged from 23% to 48% in 2021; 14% to 29% in 2022; 8 to 31% in 2023; and 12 to 55% in 2024. To ensure high rot levels for evaluating treatment efficacy, we tested three spray regimes, whereas growers in Massachusetts typically use 4-5 fungicide applications for fruit rot management. Rot levels ranging from 3 to 95 were reported in three spray regime fungicide trials on ‘Early Black’ cultivar over the years (Caruso, 1999; 2000; 2002a; 2002b; 2003; 2004; 2005; 2010; 2011), and 4 to 90% on ‘Ben Lear’ cultivar (Caruso, 2008, 2009) from MA before. No literature was found on rot levels for the ‘Demoranville’ cultivar. The differences between years likely stem from variations in weather. The current study did not investigate the effects of cranberry cultivar on rot levels comprehensively, as treatments were evaluated on ‘Demoranville’ in 2021 and 2023, while on ‘Ben Lear’ and ‘Stevens’ were evaluated for only one year each. In 2021, fruit rot levels were notably higher. Even the best treatment resulted in 23% rot. Many growers also reported increased fruit rot that year, which was likely due to the exceptionally wet conditions in early September. September 2021 experienced 279 mm of total precipitation, far exceeding the average of 117 mm (Visual Crossing). In contrast, September of 2022, 2023 and 2024 recorded 83, 159, 110 mm of total precipitation.). Cranberry fruit rot is positively correlated with increased rainfall, particularly late in the season when the fruits are mature. Excessive rainfall stresses the fruit, making it softer and more susceptible to disease spread (Shear et al., 1931; Wells and McManus, 2013).

These results highlight the potential role of these novel active ingredients, which were previously unregistered for cranberry, in managing CFR. They also emphasize the enhanced efficacy of these fungicides when tank-mixed with azoxystrobin, leading to better fruit rot control and enhanced yields. The synergistic effects of azoxystrobin with other fungicides have been noted in previous studies, such as its combination with difenoconazole (Bhuvaneswari and Krishnam Raju, 2012), propiconazole (Uppala and Zhou, 2018) against rice sheath blight, and with difenoconazole and tebuconazole against squash powdery mildew (Noshy et al., 2019). Given the complex nature of fruit rot and the wide spectrum of fungi involved, combining fungicides with different modes of action helps reduce selection pressure on pathogens and thus the development of resistance.

In conclusion, the findings from the current study demonstrate the potential of novel fungicides from FRAC groups 7, 9, and 12 to diversify the CFR management toolkit and their synergistic effects when combined with azoxystrobin.

## Data Availability

The original contributions presented in the study are included in the article/supplementary material. Further inquiries can be directed to the corresponding author.
